# The interrelationship between physical activity intensity, cardiorespiratory fitness, and executive function in middle-aged adults: An observational study of office workers

**DOI:** 10.3389/fpubh.2022.1035521

**Published:** 2022-11-09

**Authors:** Rui Wang, Maria M. Ekblom, Daniel Arvidsson, Jonatan Fridolfsson, Mats Börjesson, Örjan Ekblom

**Affiliations:** ^1^Department of Physical Activity and Health, The Swedish School of Sport and Health Sciences, Stockholm, Sweden; ^2^Division of Clinical Geriatrics, Department of Neurobiology, Care Sciences and Society, Karolinska Institute, Solna, Sweden; ^3^Wisconsin Alzheimer's Disease Research Center, University of Wisconsin School of Medicine and Public Health, Madison, WI, United States; ^4^The Department of Neuroscience, Karolinska Institute, Solna, Sweden; ^5^Center for Health and Performance, Department of Food and Nutrition, and Sport Science, Faculty of Education, University of Gothenburg, Gothenburg, Sweden; ^6^Center for Health and Performance, Department of Molecular and Clinical Medicine, Sahlgrenska Academy, University of Gothenburg, Gothenburg, Sweden; ^7^Sahlgrenska University Hospital/Östra, Gothenburg, Sweden

**Keywords:** physical activity intensities, cardiorespiratory fitness, executive function, office workers, active aging

## Abstract

**Background:**

Previous evidence supports a beneficial effect of physical activity on executive function across the whole lifespan. Yet, the interrelationships of the intensities of physical activity, cardiorespiratory fitness, and executive function require further investigation in adults.

**Aim:**

Using unfiltered accelerometry data and high-resolution intensity classification, we sought to estimate the associations of physical activity with cardiorespiratory fitness and executive function in adult office workers.

**Methods:**

We included 343 full-time office workers (mean age: 42.41 years, range of age: 36−49 years). Executive function was assessed using Stroop, Trail making tests (part-B), and 2-back tests, and a composite score was produced to reflect the general executive function performance. Physical activity was assessed using the Actigraph GT3X+-monitor, worn by each participant for seven days at the hip. Raw accelerometry data were processed by the 10 Hz frequency extended method and divided into 22 intensity bins and sleep time. Cardiorespiratory fitness was estimated using the submaximal Ekblom-Bak cycle ergometer test. Data were analyzed using partial least squares regressions.

**Results:**

In adults, cardiorespiratory fitness was closely correlated with a wide range of absolute physical activity intensity patterns. A higher level of executive function in adults was associated with both higher absolute physical activity intensities and cardiorespiratory fitness, which was independent of age, sex, and education levels. A very weak association between intensities, fitness, and executive function was observed in high-fit adults. Among low-fit adults, although a positive association started already toward the upper end of moderate intensity, there still appeared to be an association between intensities, cardiorespiratory fitness, and executive function. That is, cardiorespiratory fitness may mediate the association between absolute physical activity intensities and executive function up to a certain level.

**Conclusion:**

The maintenance of executive function in adulthood was related to both physical activity intensities and cardiorespiratory fitness, while their interrelationship was not equal across fitness levels. It is highly recommended to consider the cardiorespiratory fitness level in future studies that focus on executive functions in aging as well when designing individualized physical activity training programs.

## Background

Executive function, as a set of cognitive processes, is related to higher-order of cognitive processes (e.g., planning, working memory, and inhibiting) and goal-directed behaviors (1, 2). Aging preferentially affects certain cognitive domains more than others, and a decline in executive function is one of the hallmarks of cognitive aging (3, 4). Although the decline in executive function is partly attributable to the anatomical changes of the brain areas (e.g., frontal lobe atrophy), it remains unclear why individual differences exist in executive function, especially in relation to the aging process (4–6).

The beneficial effect of physical activity on cognition, including executive function, has been recognized in early and late periods of lifespan, as well as in certain populations characterized by cognitive deficits, e.g., Alzheimer's disease (7–13). Yet, a limited body of evidence supports the link between physical activity and executive functioning in young to middle-aged adults (14). A recent systematic review displays that among sedentary adults without cognitive impairment, aerobic physical activity interventions may preserve executive function with a small amount of effect (15). Considering that the selection criteria of participants in physical activity interventions may be strict in certain groups, observational studies are thus necessary to explore the link between physical activity intensity levels and executive function among adults in a deeper manner (9).

Both chronic and acute effect on executive function from physical activity has been presented previously. For instance, acute bouts of moderate-to-vigorous physical activity exhibit a temporal benefit on executive function during the post-recovery period following exercise, which is shown mostly in studies with children (16–18). The chronic effect is indicated by the greater effectiveness of physical activity intervention on cognitive outcomes seen in studies with longer intervention lengths (19). It is uncertain how the intensity of physical activity is linked to executive function across adulthood. Firstly, physiological reactions to physical exercise vary foremost with its intensity, such as increases over resting values in the cerebral circulation, lactate concentration, and secretion of neurotrophines (20, 21). Evidence is lacking to draw a holistic picture between a wide spectrum of physical activity intensity and executive functioning in adults. Secondly, the magnitude of the physiological reactions to physical activity, especially to acute exercise, is normally relative to the maximally tolerated work capacity (termed as cardiorespiratory fitness) (22). It differs greatly between individuals, with a 2–3-fold variation in the mixed populations (23, 24). Individuals with higher levels of physical exercise and cardiorespiratory fitness during midlife have displayed better-preserved cognition in late life, (25) but little is known about the interrelationship among the three domains in earlier lifespan.

**Figure 1:**
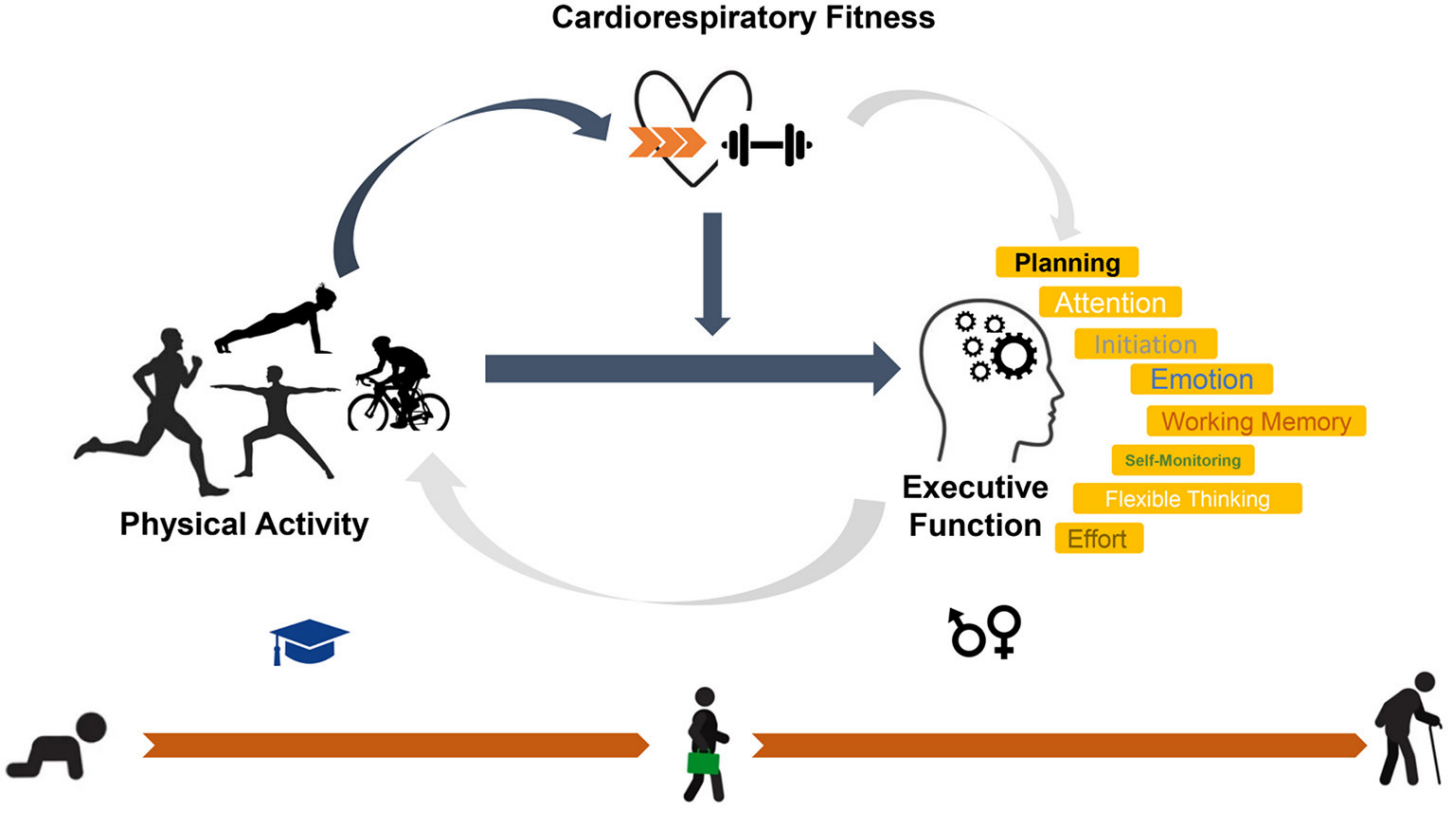
Theoretical model of a lifelong interrelationship between physical activity, cardiorespiratory fitness, and executive function. The working model hypothesized that different physical activity intensities may slow cognitive aging in the subdomain of executive function, from middle age through late life. Sufficient physical activity intensity may preserve executive functioning in middle age through its effect on cardiorespiratory fitness or other neuroprotective pathways. Moreover, we theorized that cardiorespiratory fitness may moderate the association between specific physical activity intensity patterns and executive function. We will investigate thoroughly the interrelationship between physical activity intensities, cardiorespiratory fitness, and executive function by following the pathways pointed by dark arrows in this figure. The gray arrows indicate (1) possible pathways from cardiorespiratory fitness to executive function, such as cerebral perfusion and cardiovascular health, (2) reverse pathways from executive function to physical activity intensities, such as making exercise plans.

In the current study, our working model hypothesized that different physical activity intensities may show association to various degrees of slower cognitive aging, in the subdomain of executive function, from middle age through late life. Physical activity of sufficient intensity may preserve executive functioning in middle age through its effect on cardiorespiratory fitness or other neuroprotective pathways. Moreover, we theorized that cardiorespiratory fitness may moderate the association between specific physical activity intensity patterns and executive function (Figure 1). In this study of middle-aged office workers, we aim to thoroughly investigate the interrelationships between physical activity intensity spectrum, cardiorespiratory fitness, and executive function, *via* applying device-based physical activity measurements with more than 20 intensity classes.

## Methods

### Participants

The data in this study were retrieved from a larger project with a cross-sectional design called Physical Activity and Healthy Brain Functions (data collection: Jan 2016— Dec 2017) (26). Study participants were selected from the office-based employees at three workplaces in Stockholm and Gothenburg. In total, 2024 employees were invited to participate in this study and to respond to a self-reported web-based questionnaire. Approximately 1 week after the online survey has been completed, participants were invited to take part in an assessment of executive function using computer-based and pen-and-paper-based tests as well as starting their week-long physical activity assessment. The Stockholm regional ethical review board approved the project (Dnr 2016/1840-32) and all participants signed a written informed consent form.

### Executive functions testing

A comprehensive cognitive test battery (9 tests: 6 were computerized tests and 3 were with pen and paper) was administered at the workplaces during office hours (test duration: ~45 min) by trained test leaders. We applied E-Prime 2.0 (Psychology Software Tools) for computerized tests. All cognitive tests were conducted in a specific order, which have shown good validity and reliability for measurement of the respective cognitive domains. Details of these cognitive tests were described previously (26).

Executive functions were measured by Trail Making Test-B (TMT-B, shifting), 2-back (updating), and the Stroop test (inhibition) (27). TMT-B had 25 encircled, but with digits and letters that were to be connected in alternating order (1-A-2-B-3-C, etc.). The outcome score for 2-back was accuracy for 4 blocks of 20-digit sequences. The Stroop paper test consisted of 50 incongruent printed color words (5 rows with 10 words, e.g., the word blue printed in red color). A practice of 10 words was completed prior to the test. During the test, the test leader commented on errors, and participants needed to say the correct color (not read the word) for all targets until the test ended. The outcome score was the completion time.

The reverse scores of TMT-B and Stroop were estimated by calculating the distance between the maximum value and individual scores. A standardized score was further calculated for TMT-B reverse score, Stroop reverse score, and 2-back accuracy score, respectively. We calculated the average score of the three standardized scores as a composite score of executive function, with a higher score indicating a better function.

### Physical activity assessments

#### Device-based data collection

After executive function testing, participants were equipped with an activity monitor (ActiGraph GT3X, Pensacola, FL, USA) and received a diary logbook. The participants were asked to wear the activity monitor during all waking time (except for water-based activities) and noted the time points for when they went to bed and woke up, for seven consecutive days. The accelerometer was worn on the right hip with an elastic band. Accelerometers were set to record at a sampling frequency of 30 Hz. To be considered valid and included in the analyses, recordings were required to cover at least 600 min per day on at least four separate days (28).

#### Analysis/processing of device-based physical activity data

We applied a filter with a frequency range of 0.29–10 Hz to process the raw accelerometry data on physical activity intensity (29, 30). We defined the non-wearing time as a continuous zero output for at least 60 min with an allowance of up to 2 min of output above zero but below the sedentary cut-point. Time to bed or sleep time was identified according to the daily diary records from the participants during the measurement period. When there are missing records, the standard time in and out of bed was replaced as 23:00 and 06:00, respectively. The physical activity intensity of the time defined as sensor worn and out of bed was divided into specific intensity levels— it is based on accelerometry output cut-points in two ways (as described below). To describe the activity patterns, worn time was divided into sedentary, light-, moderate-, vigorous- and very-vigorous physical activity, using counts-based physical activity data, referring to 1.5, 3, 6, and 9 metabolic equivalents of tasks (METs), respectively. To provide more detailed analyses of the physical activity intensity range, it was divided into a spectrum of 22 smaller bins, using filtered raw acceleration data (expressed in milli-gravity units, mg). The width of the bins was chosen to ensure (1) having enough detail at low intensity with the MET cut-points, and (2) avoiding extreme detail at high intensities. The bin edges were 0, 40, 80, 160, 240 mg, and so forth, increasing with 80 mg (30). The physical activity intensity level of time out of bed and sensor not worn on valid days was extrapolated assuming an equal distribution as the time the sensor was worn. Accelerometry data processing was performed in MATLAB R2020a (MathWorks, Natick, MA, USA).

### Fitness testing

The submaximal Ekblom-Bak cycle ergometer test was performed to collect the data on estimated cardiorespiratory fitness (31). During a sub-maximal cycle ergometer test, the heart rates that responded to different sub-maximal rates of work were recorded using telemetry (Polar Oy, Tampere, Finland). We estimated cardiorespiratory fitness, or maximal oxygen consumption, using the sex-specific equations based on the difference in heart rate response between two different sub-maximal rates of work, i.e., a standardized work rate and a higher, individually set rate of work. In our analysis, fitness was expressed as a relative value (milliliters per minute per body mass). It was calculated using a revised algorithm which has been shown to give a valid and reliable estimate of the directly measured maximal oxygen consumption (r = 0.90) in adults (32).

### Statistics

Characteristics of subjects were described by gender using either mean (standard deviation, SD) or median (interquartile range, IQR) for continuous variables, and frequecies (percentage) for categorical variables. We compared differences in characteristics between men and women using a two-sample *t*-test or Mann-Whitney U-test for continuous variables, and a chi-square test for categorical variables. We standardized the intensity of the physical activity spectrum and sleep to z-scores for further data analysis.

Three steps of analysis were carried out to investigate the interrelationships between physical activity intensity, cardiorespiratory fitness, and executive function. In the first step of the analysis, we aim to determine the association between physical activity intensity and cardiorespiratory fitness. In the second step, we aim to verify the combined contribution of physical activity intensity and cardiorespiratory fitness to executive function. In the third step of the analysis, we aim to determine whether the link between physical activity intensity pattern and executive function varies by cardiorespiratory levels.

Because the data on the physical activity spectrum are highly correlated with each other (correlations up to 0.97), in the first and second steps of analysis, we applied partial least squares (PLS) regression to explore the association between the spectrum of physical activity, cardiorespiratory fitness, and executive function. Briefly, PLS regressions estimate the associations between the components of independent variables (intensity of physical activity/cardiorespiratory fitness) and response variable (cardiorespiratory fitness/executive function). The number of components was chosen by the smallest value of Root Mean Square Error (RMSE) in the cross-validation. We applied permutation tests to reflect the importance of the selected PLS model, and an alpha value was reported. If the alpha value is quite small, it suggests that the covariance for unpermuted data should be larger than the covariance for permuted data, indicating a probability that the given model is significantly different from one built under the same conditions but on random data. The β-coefficients and their 95% confidence intervals (CI) of independent variables in each PLS regression model were reported to reflect the contribution of predictors to the shared components as well as the associations between independent variables and response variables. To fully understand the relationship of physical activity patterns and cardiorespiratory fitness with executive functions in the second step of the analysis, we performed different PLS models to consider the confounding effects of other factors (i.e., age, gender, education). We first ran a PLS model using executive function as the outcome, and physical activity intensity and cardiorespiratory fitness as predictors. The second model was conducted using the same predictors, but we removed the effect of age, gender, and education status from the outcome of executive function. This was done using the residuals from a multiple linear regression with age, gender, and education as independent variables and the executive function as the dependent variable (33).

In the third step of the analysis, to entirely understand whether certain patterns of physical activity intensity are associated with executive function and whether the patterns are independent of cardiorespiratory fitness, PLS-regression models were performed (predictor: physical activity intensities, outcome: executive function), stratifying by gender-specific low- and high-fitness groups.

To further understand the interrelationships between physical activity intensity, cardiorespiratory fitness, and executive function, in the additional analyses, we divided our study participants into four groups according to their joint executive function and cardiorespiratory fitness— (1) low-fit and low executive function, (2) low-fit and high executive function, (3) high-fit and low executive function, and (4) high-fit and high executive function. We described the average level of weekly time spent on different activities in the four groups.

Our analysis was performed using R 4.0.5 software and STATA 16.0 (STATA Corporation, College Station, TX).

## Results

### Characteristics of study participants

In total, 343 participants (233 women and 110 men) with available physical activity data, were included in the analyses (Table 1). The average age of study participants was 42.4 ± 9.0 years and the average length of education was 14.4 years. Compared with women, men showed higher levels in education year, cardiorespiratory fitness, and sedentary time, whereas less time was spent on moderate intensity physical activity. No significant differences were detected between men and women regarding age, executive function, wear time, low-intensity physical activity, vigorous-intensity physical activity, and very vigorous intensity physical activity.

**Table 1:** Characteristics of study participants (*n* = 343).

	**Total (*n* = 343)**	**Gender**		
		**Women (*n* = 233)**	**Men (*n* = 110)**	***P*-value**
Age (years), mean/SD	42.4 (9.0)	41.8 (9.3)	43.41 (8.4)	0.159
Education (years)^a^ mean/SD	14.4 (2.3)	14.2 (2.3)	14.85 (2.2)	0.012
Cardiorespiratory fitness (mL/kg×min)^a^, mean/SD	39.9 (8.4)	37.7 (7.8)	44.56 (7.7)	<0.001
Executive function^a^, mean/SD	0.01 (0.7)	0.03 (0.8)	−0.03 (0.7)	0.535
**Accelerometry data, median (IQR)**				
Valid days	8.0 (7.0–8.0)	8.0 (7.0–8.0)	8.0 (7.0–8.0)	0.839
Average wear time per day (minutes)	960.81 (926.9–988.8)	960,0 (927.5–985.0)	968.6 (926.9–1010.6)	0.078
Average sleep time (minutes)	479.2 (451.3–513.1)	480.0 (455.0–512.5)	471.4 (429.4–513.1)	0.078
Average time spent sedentary (minutes)	751.2 (716.4–798.0)	745.4 (716.2–788.8)	762.9 (721.5–819.4)	0.039
Average time spent in low-intensity PA (minutes)	116.7 (99.4–137.2)	115.2 (99.5–137.5)	120.0 (99.4–135.6)	0.68
Average time spent in moderate intensity PA (minutes)	76.1 (65.3–92.1)	79.2 (67.2–94.4)	72.7 (59.1–81.5)	<0.01
Average time spent in vigorous intensity PA (minutes)	1.4 (0.6–3.8)	1.5 (0.7–3.9)	1.1 (0.5–3.3)	0.12
Average time spent in very vigorous intensity PA (minutes)	0.1 (0.0–0.4)	0.1 (0.0–0.3)	0.1 (0.0–0.7)	0.70

a There were two missing values for education, 5 for cardiorespiratory fitness level, 51 for executive function score.

SD, Standard Deviation; IQR, Interquartile range; PA, Physical Activity.

### Association between physical activity intensity and cardiorespiratory fitness

**Figure 2:**
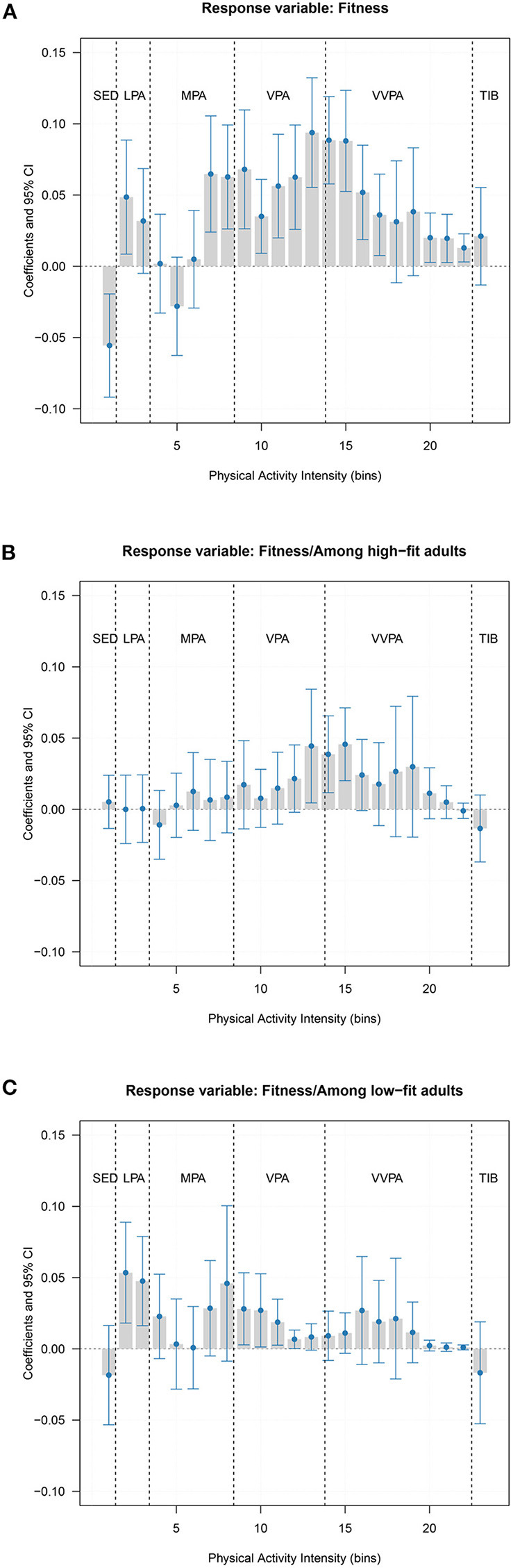
Association between physical activity intensity and cardiorespiratory fitness. A positive bar indicates more time spent at that activity intensity is associated with a higher level of cardiorespiratory fitness, while a negative bar indicates more time spent at that activity is associated with a lower level of fitness. SED, Sedentary; LPA, Light physical activity; MPA, Moderate physical activity; VPA, Vigorous physical activity; VVPA, Very vigorous physical activity; TIB, Time in bed/sleep time. **(A)** Displays the results in all analytical sample, **(B)** shows the results in the high-fit adults, and **(C)** presents the results in the low-fit adults.

Based on the smallest RMSE value (see Supplementary Figure 1A), we selected one component in the PLS regression (18.39% of variance explained in the cross-validation) to associate cardiorespiratory fitness (response variable) with the physical activity spectrum. Permutation test showed that the PLS model linking physical activity intensity to cardiorespiratory fitness in all participants was significant (alpha value<0.001). This, together with the β-coefficients (95% CI) of the physical activity spectrum from the PLS regression (Figure 2A), indicated that cardiorespiratory fitness was associated with a physically active lifestyle pattern (i.e., limited sedentary time and more time spent in activities with a wide range of intensity levels). We further applied the gender-specific median value of cardiorespiratory fitness (44.4 mL/kg × min for men and 37.40 mL/kg × min for women) to categorize our participants into high-fit and low-fit groups. The associations between cardiorespiratory fitness and physical activity intensity spectrum seemed to vary by the fitness levels. When the stratification analyses (Figures 2B,C) were carried out by the fitness groups, we found that the PLS regression was significant among low-fit adults (alpha value = 0.04) but not among high-fit adults (alpha value = 0.09).

### The contribution of physical activity intensity and cardiorespiratory fitness to executive function

**Figure 3:**
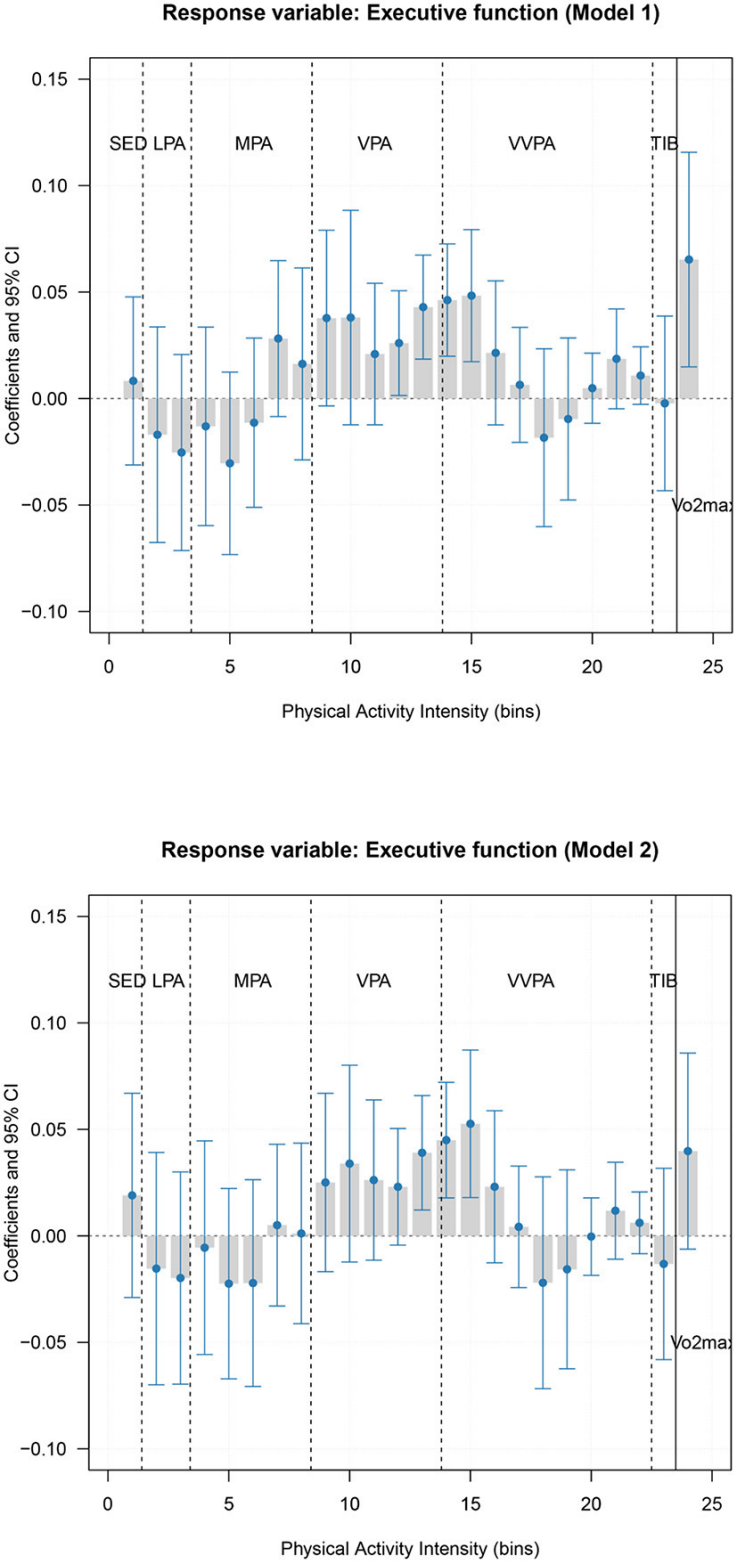
Association of physical activity intensity variable (bin) and cardiorespiratory fitness with the executive function composite score. A positive bar indicates more time spent at that intensity is associated with higher executive function, while a negative bar indicates more time spent at that activity is associated with lower executive function. The last bar marked with Vo2max reflects the association between cardiorespiratory fitness and executive function. Model 1 is unadjusted model, and Model 2 was additionally adjusted for the influence of age, sex, and education level. SED, Sedentary; LPA, Light physical activity; MPA, Moderate physical activity; VPA, Vigorous physical activity; VVPA, Very vigorous physical activity; TIB, Time in bed/sleep time.

When we linked physical activity intensity and fitness to executive function, both unadjusted and adjusted models suggest the PLS regression model with one component (16.3% of the variance was explained in the unadjusted model, and 15.4% of the variance was explained in the age, sex, and education adjusted model, see Supplementary Figures 1B,C). Results in the unadjusted PLS regression model (Figure 3, Model 1) displayed that higher executive functions were associated with both more time spent in vigorous and very vigorous physical activity as well as a high level of cardiorespiratory fitness, although the PLS model was not significant (alpha value = 0.13). A similar weekly activity pattern was demonstrated in the adjusted PLS regression model (Figure 3, Model 2).

### The link between physical activity intensity and executive function by cardiorespiratory fitness levels

**Figure 4:**
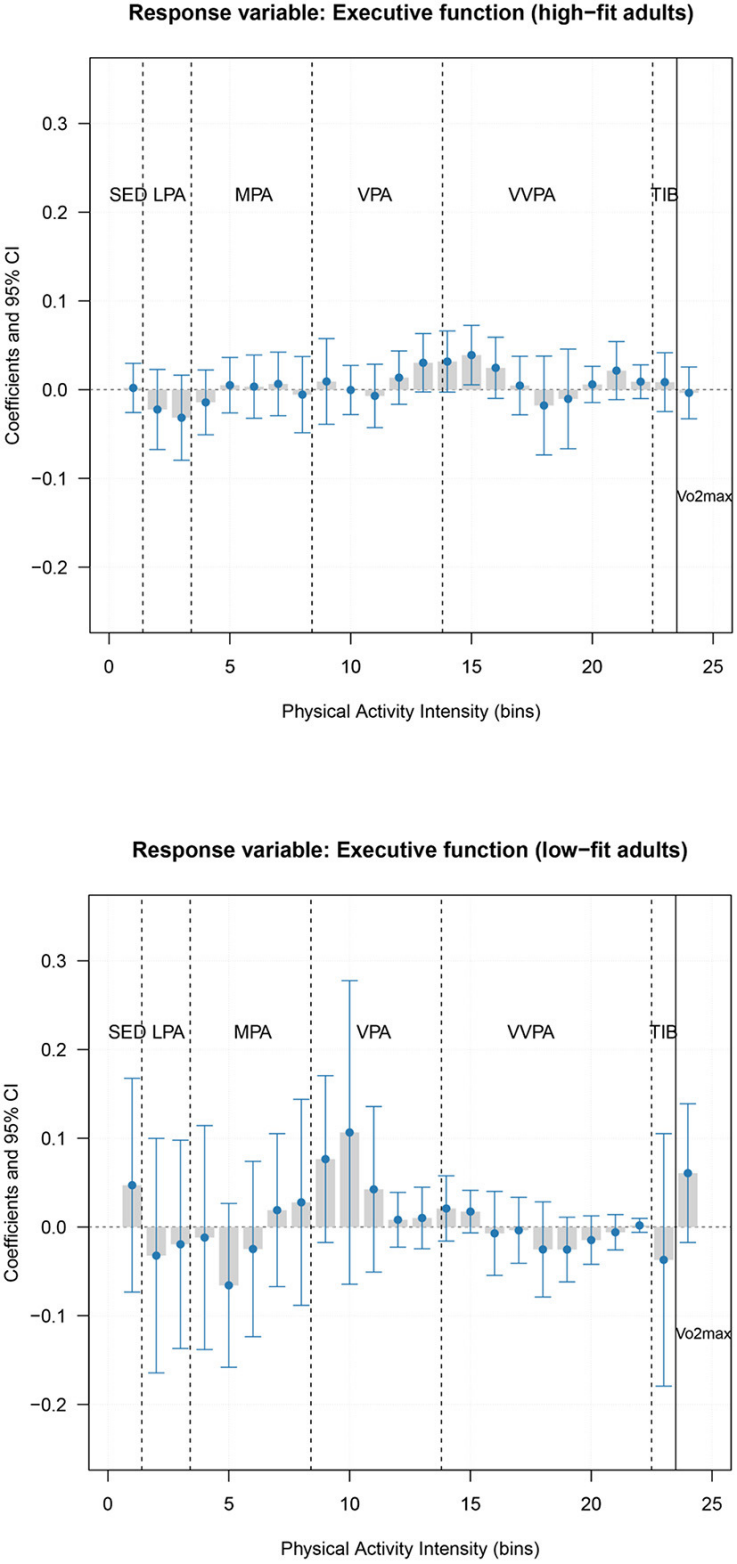
Association of physical activity intensity variable (bin) and cardiorespiratory fitness with the executive function composite score by fitness level. The last bar marked with Vo2max reflects the association between cardiorespiratory fitness and executive function. We applied gender-specific cut-off value of cardiorespiratory fitness to categorize our participants into high-fit (men: Vo2max>44.56 mL/kg × min; women: Vo2max>37.69 mL/kg×min; *n* = 143) and low-fit (men: Vo2max≤44.56 mL/kg/min; women: Vo2max≤37.69 mL/kg/min; *n* = 145) groups. SED, Sedentary; LPA, Light physical activity; MPA, Moderate physical activity; VPA, Vigorous physical activity; VVPA, Very vigorous physical activity; TIB, Time in bed/sleep time.

To understand whether the association between physical activity intensity and executive function was independent of cardiorespiratory fitness level, we further conducted the stratified PLS regression model by cardiorespiratory fitness levels. Supplementary Figures 1D,E showed the RMSE values for the selected PLS regression model in high-fit adults and low-fit adults, respectively. Although permutation tests suggested that PLS models in both high-fit and low-fit adults were not significant (alpha value _high−fit_ = 0.95 and alpha value _low−fit_ = 0.39), Figure 4 displayed that the relationships of physical activity intensity and cardiorespiratory fitness with executive function may differ between high-fit and low-fit adults. Among high-fit adults (*n* = 143), only weak associations were observed between physical activity, cardiorespiratory fitness, and executive function. Yet, among low-fit adults (*n* = 145), a higher executive function tended to relate to more time spent sedentary, and in vigorous physical activity (although a positive association started already toward the upper end of moderate intensity), as well as a higher level of cardiorespiratory fitness. The same results were obtained when we excluded cardiorespiratory fitness in the PLS model (Supplementary Figure 2).

### Additional analyses

Results in the additional analyses showed that there were no substantial differences in light-to-moderate physical activity intensities between the four joint groups. Yet, compared to low-fit adults, high-fit adults presented less sedentary time and more time in bed/sleep time, which was regardless of executive function level. More differences between the four joint groups were displayed in the high levels of physical activity intensities. That is, the group with low-fit and low executive function demonstrated the least time spent in vigorous to very vigorous physical activity, whereas the group with high-fit and high executive function appeared to show the most time spent on these activities.

## Discussion

### Main findings

Findings from the current study can be summarized as (1) absolute physical activity intensity and cardiorespiratory fitness may have an interdependent effect on mid-life executive function; (2) the joint effect of absolute physical activity intensity and cardiorespiratory fitness on executive function among adults may be largely diluted when cardiorespiratory reached to certain level.

### In comparison with other studies

Previous research only shows a general relation between physical activity and executive functions, especially in children (34). One possible reason for the lack of detailed physical activity intensity data is that only a few previous studies have applied sensor-based measurements of physical activity, as self-reported data lack precision and temporal resolution. In most studies that engaged sensor-based physical activity measurements, physical activity data is often processed into three or four intensity classes using the cut-point of ActiGraph counts-based energy expenditure, with a class width span normally varying between 1.5 METs and infinity for sedentary behavior, 1–1.5 METs for light-intensity physical activity, 1.5–3 METs for moderate-intensity physical activity, and 3–6 METs for vigorous physical activity, 6–9 METs for very vigorous physical activity (35). This may narrow down the physical activity intensity spectrum and loss much information regarding the accelerometry data (30). Further, in the most frequently used monitor, the ActiGraph GT3X, a frequency filter, has been applied. This may limit the investigations of activities inducing high frequent accelerations, and thus hinder detailed investigations on the relation between vigorous physical activity and executive functions.

Most previous studies that investigated the association between physical activity and executive function did not take variations of cardiorespiratory fitness into account. This may be of importance, as health effects are associated with relative intensity, not absolute physical activity intensity. It may, therefore, well obscure relations between activities of different intensities and cognitive performance. Thus, taking fitness into account, for example *via* stratified analyses, is an important step in expanding our understanding of the relations between physical activity and cognitive abilities. In our stratified analyses, different patterns were observed between high-fit and low-fit adults regarding the association of physical activity intensity and cardiorespiratory fitness with executive function. Notely, accelerometry data capture absolute intensity of physical activity but the output measurement is often expressed in relative terms (i.e., time spent in moderate or vigorous physical activity). Relative physical activity intensity is dependent on individual levels of fitness, which itself might be influenced by multiple factors, e.g., genes and health conditions. Thus, the relationships between relative activity intensities and executive function may actually show similar patterns between high-fit and low-fit adults, though the patterns of absolute physical activity differ. More research is required to further explore the connections between relative physical activity intensities, fitness, and executive function. In addition, the unsignificant PLS models and β-coefficients in the stratified analyses might be attributable to the fact that stratifications remove a lot of the variation and leaves less statistical power to locate significant associations.

Although the beneficial effect of physical activity on cardiorespiratory fitness measured by VO_2_max has been well documented by previous studies, (36, 37) findings remain inconclusive regarding the association of physical activity intensities with cardiorespiratory fitness, especially in adults. Studies based on self-reported physical activity data have provided a general link between physical activity and VO_2_max. For example, a study involving adolescents who were followed for 23 years untill adulthood revealed that self-reported physical activity level is weakly related to VO2max (38). A study involving German women showed that a self-reported high level of leisure time activity, was associated with high VO_2_max (39). Studies based on device-based physical activity measurements have mostly been focusing on certain physical activity patterns, such as the association of VO_2_max with moderate-to-vigorous physical activity (40). Our findings, although from a cross-sectional design, suggest that physical activity of lower absolute intensities was sufficient to improve cardiorespiratory fitness even in low-fit adults, which differed from high-fit adults where physical activity of higher intensities was required.

The interrelationship between physical activity, cardiorespiratory fitness, and cognitive performance has been investigated essentially in children and older adults, (41, 42) but is understudied in observational studies involving healthy adults. A recent study involving young adults has provided the first neural evidence that cardiorespiratory fitness levels modify the effect of acute exercise on executive function (43). To the best of our knowledge, this is the first work that thoroughly examined the interrelationship between absolute physical activity intensity, cardiorespiratory fitness, and executive function in healthy adults. Our findings have highlighted that the relation between different absolute physical activity intensities and executive functions through adulthood varied with fitness levels.

### Mechanisms and interpretations

Although habitual high-intensity exercise generally increases aerobic fitness, this investigation could not confirm that fitness is independently linked to higher executive functions. Nevertheless, cardiorespiratory fitness level may serve as a mediator or moderator to explain enhanced cognitive performance through different levels of intensity training. In general, moderate and vigorous intensity exercises can raise VO2max levels, increase oxygen delivery to the working muscles, and facilitate higher oxygen extraction in the muscles. Yet, the individual fitness level does influence the optimum absolute intensity needed for sufficient stimulus. Our findings are in line with previous studies showing that compared to low-fit adults, well-trained adults require higher absolute intensities to gain improvements in their cardiorespiratory fitness (44). The finding that cardiorespiratory fitness moderated the associations between absolute physical activity intensities and executive function so that higher absolute intensities related to executive function in higher fit as compared to lower fit individuals, supports the notion that the intensity of exercise prescribed to improve executive functions should be set relative to the fitness of the individual. Besides the moderating role, cardiorespiratory fitness may act as a mediator linking physical activity to executive function. The influence of cardiorespiratory fitness on cognition may be through cerebral blood flow regulation or a molecular pathway beyond vascularization, such as N-acetyl aspartate (45, 46). Further, a threshold of VO2max may exist with no further effect of physical activity on executive function, as indicated by our stratified analyses with only weak association in high-fit individuals but with stronger associations in low-fit individuals. However, additional research is required to confirm our non-significant findings and to explore other pathways between physical activity intensities and executive function that are moderated but not mediated by fitness.

### Strength and limitations

Several strengths are included in the current study. Firstly, calibration research has revealed that the ActiGraph counts become insensible at higher physical activity intensities, such as vigorous physical activities, in comparison with walking (47). With the improvement of accelerometry raw data processing, we have applied a wider frequency filter, which has been shown to better capture the variations in physical activity and movement patterns (29). Secondly, to avoid the limitation of cut points, we divided the intensity spectrum into many blocks or bins, which keeps much more accelerometry information and improves the knowledge about the association pattern of physical activity. Thirdly, applying multivariate analysis, i.e., PLS regression, can handle well the collinear nature of the intensity spectrum of physical activity variables as shown by large proportion of high correlation. We applied PLS regression to identify the potential relations between a matrix of physical activity variables, cardiorespiratory fitness, and executive function, which were assessed by the explained variance, the magnitude of coefficients, and model fitness. Fourth, applying device-based measurements in this study (i.e., physical activity and cardiorespiratory fitness) can overcome the reporting bias that often exists in epidemiological studies with questionnaire data. Several limitations likewise remain in our study. With the cross-sectional design, the bidirectional association between physical activity pattern and cardiorespiratory fitness makes it difficult to distinguish the long-term influence of physical activity pattern on fitness and executive function as well as the causal effects. Lacking relative intensity measurements, we were unable to compare the patterns between relative and absolute intensities in relation to both fitness and executive function. Large variation may exist in physical activity data regarding the investigated intensity levels. The habitual physical activity pattern may not be captured entirely using a 7-day of physical activity measurement (48). There might be a small effect of valid day and wearing time on our study results, although previous research did not observe much difference (30). In this study, we included 343 participants, 338 were with available data on cardiorespiratory fitness and 292 were with available data on executive function. Results in the permutation tests showed that only the PLS model linking physical activity intensity and cardiorespiratory fitness in all participants (*n* = 338) was significant (alpha value<0.001). This indicates that the PLS analysis of physical activity patterns and executive function is plausible to have an insufficient power of estimation owing to the limited number of available participants. In addition, volunteered participants in our study are more likely to show a profile of well-educated, highly motivated, and active office workers. Although tendencies of association between low-intensity of physical activity pattern, fitness, and executive function were observed in our results, cautions are needed when generalizing the findings to other populations. Another limitation of the current study is lacking objective measures of cardiovascular health and lung function. The interrelationships between physical activity intensities, cardiorespiratory fitness, and cognition may differ by these factors.

## Conclusion

The maintenance of executive function in adulthood is related to both physical activity intensities and cardiorespiratory fitness, while their interrelationship may be fluctuated by the cardiorespiratory fitness. Firstly, to increase or maintain cardiorespiratory fitness in adults, training programs should be adapted to the cardiorespiratory fitness level, such as the absolute physical activity intensity level should be gradually increased to enhance cardiorespiratory fitness. In this way, less fit individuals are able to achieve a higher fitness level with less (absolute) intensity work. Secondly, cardiorespiratory fitness may mediate the effect of physical activity on executive function up to a certain level. Future research that focuses on exercise-related cognitive boosting in aging thus is recommended to take cardiorespiratory fitness level into account. More investigations are necessary to explore other pathways linking physical activity intensities to cognitive maintenance, such as neuroplasticity.

## Data availability statement

Derived and raw data supporting the findings of this study are available from the corresponding author on request.

## Ethics statement

The studies involving human participants were reviewed and approved by the Stockholm Regional Ethical Review Board approved the project (Dnr 2016/1840-32) and all participants signed a written informed consent form. The patients/participants provided their written informed consent to participate in this study. Written informed consent was obtained from the individual(s) for the publication of any potentially identifiable images or data included in this article.

## Author contributions

RW and ÖE: conceptualization. RW, ME, DA, JF, MB, and ÖE: methodology and writing–review and editing. RW: formal analysis and writing–original draft preparation. ME, DA, JF, MB, and ÖE: resources. ÖE and ME: funding acquisition. All authors contributed to the article and approved the submitted version.

## Funding

This research was funded by the Knowledge Foundation (Grant Nos. 20160040 and 20210002) and by the following companies: ICA-gruppen, Intrum, SATS, Elixia, Monark Exercise, and Itrim Sweden. RW's position was supported by the Knowledge Foundation (Recruitment Grant, 20180151).

## Conflict of interest

The authors declare that the research was conducted in the absence of any commercial or financial relationships that could be construed as a potential conflict of interest.

## Publisher's note

All claims expressed in this article are solely those of the authors and do not necessarily represent those of their affiliated organizations, or those of the publisher, the editors and the reviewers. Any product that may be evaluated in this article, or claim that may be made by its manufacturer, is not guaranteed or endorsed by the publisher.
