# Supplementary material for: The interrelationship between physical activity intensity, cardiorespiratory fitness, and executive function in middle-aged adults: An observational study of office workers
Source: Front Public Health. 2022 Nov 9;10:1035521. doi: 10.3389/fpubh.2022.1035521 (PMC9682261; doi:10.3389/fpubh.2022.1035521)
Supplement: Supplementary file 1 [file Table_1.DOCX]

**SUPPLEMENTARY MATERIALS**


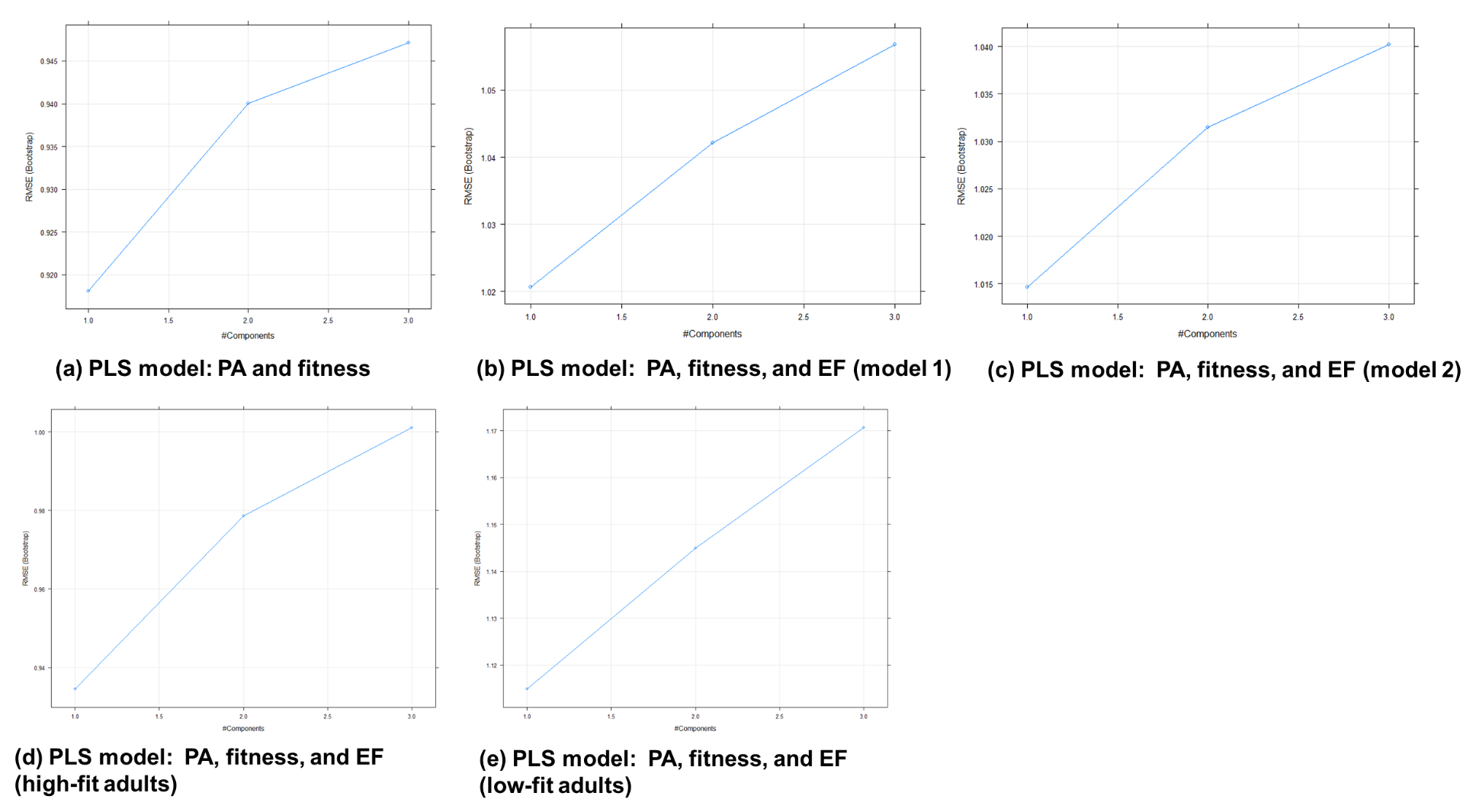


**S-Figure 1.** Values of Root Mean Square Error in different PLS regression models


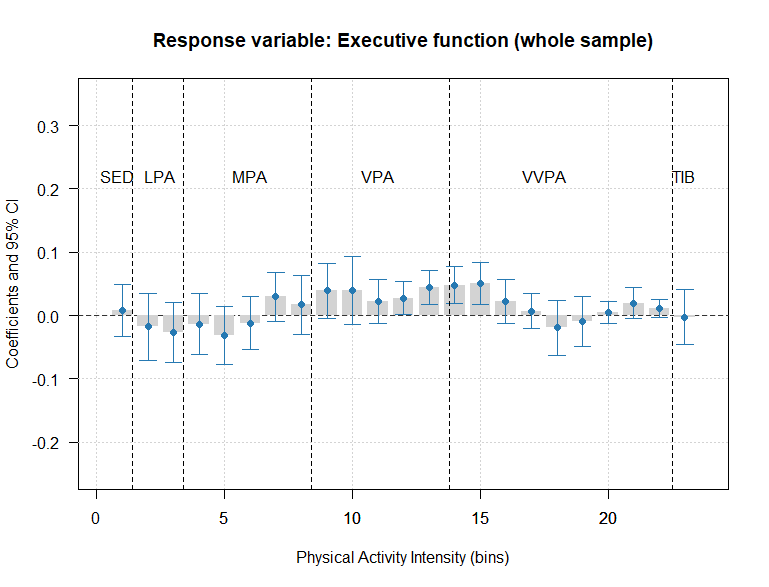

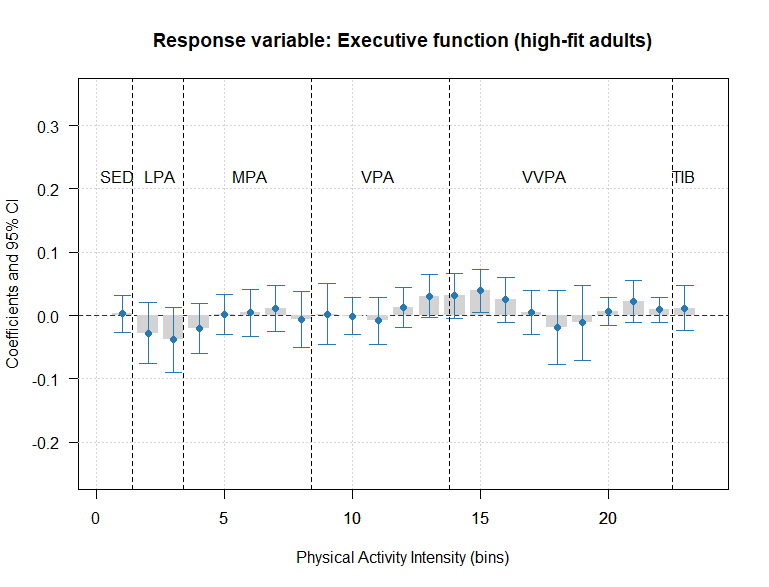

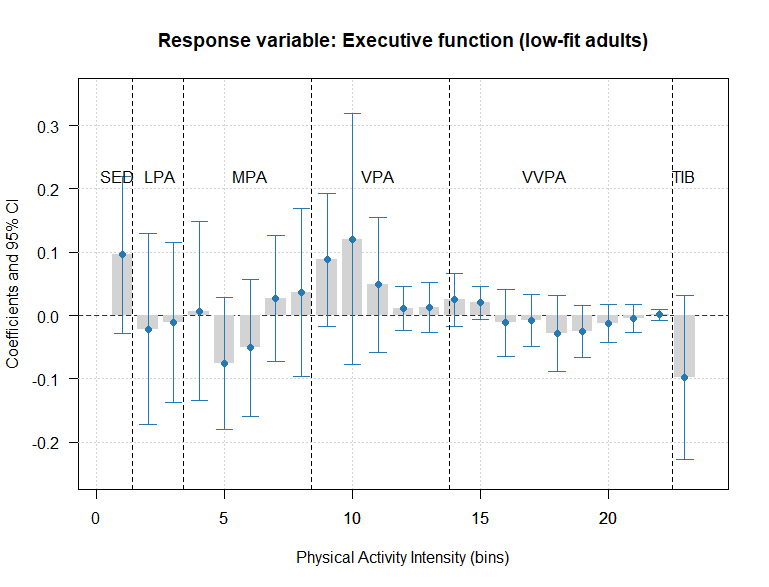


**S-Figure 2.** The association between physical activity intensities and executive function

SED=Sedentary; LPA=Light physical activity; MPA=Moderate physical activity; VPA=Vigorous physical activity; VVPA=Very vigorous physical activity; TIB=Time in bed/sleep time.

**S-Table 1. Z-score of the physical activity intensities and sleep time.**

| Variable | Obs | Mean | Std. Dev. | Min | Max |
| --- | --- | --- | --- | --- | --- |
| Physical Activity Intensities | | | | | |
| Bin 1 | 343 | 0.000643 | 1.003292 | -4.85286 | 2.802302 |
| Bin 2 | 343 | 0.004856 | 1.003623 | -2.59544 | 3.342597 |
| Bin 3 | 343 | 0.005494 | 1.005258 | -2.52889 | 3.767131 |
| Bin 4 | 343 | 0.007142 | 1.005321 | -2.64073 | 3.801643 |
| Bin 5 | 343 | 0.007505 | 1.003841 | -2.13014 | 5.339719 |
| Bin 6 | 343 | 0.001998 | 1.00245 | -1.73047 | 5.21216 |
| Bin 7 | 343 | 0.005 | 1.004057 | -1.23474 | 4.107235 |
| Bin 8 | 343 | 0.00516 | 1.00525 | -0.69265 | 9.034923 |
| Bin 9 | 343 | 0.006855 | 1.005559 | -0.72752 | 8.281922 |
| Bin 10 | 343 | 0.005006 | 1.006377 | -0.51535 | 13.15636 |
| Bin 11 | 343 | 0.0044 | 1.006592 | -0.40956 | 10.95288 |
| Bin 12 | 343 | 0.003901 | 1.006756 | -0.28418 | 14.85627 |
| Bin 13 | 343 | 0.005342 | 1.006293 | -0.38359 | 8.334879 |
| Bin 14 | 343 | 0.004896 | 1.006452 | -0.34437 | 9.986707 |
| Bin 15 | 343 | 0.004499 | 1.00658 | -0.31599 | 9.129551 |
| Bin 16 | 343 | 0.003458 | 1.006867 | -0.24562 | 10.6411 |
| Bin 17 | 343 | 0.003002 | 1.006964 | -0.22117 | 10.17238 |
| Bin 18 | 343 | 0.002319 | 1.007092 | -0.17158 | 12.85216 |
| Bin 19 | 343 | 0.001548 | 1.007108 | -0.17437 | 14.41951 |
| Bin 20 | 343 | 0.000588 | 1.007008 | -0.13552 | 17.75108 |
| Bin 21 | 343 | 7.56E-05 | 1.006644 | -0.15354 | 16.91877 |
| Bin 22 | 343 | 0.001662 | 1.007188 | -0.114 | 17.57567 |
| Sleep time | 343 | -0.00752 | 1.003201 | -3.63649 | 7.60148 |
